# Association between caffeine consumption and bone mineral density in children and adolescent: Observational and Mendelian randomization study

**DOI:** 10.1371/journal.pone.0287756

**Published:** 2023-06-29

**Authors:** Aiyong Cui, Peilun Xiao, Jing He, Zhiqiang Fan, Mengli Xie, Long Chen, Yan Zhuang, Hu Wang

**Affiliations:** 1 Department of Orthopaedics, Honghui Hospital, Xi’an Jiao Tong University, Xi’an, China; 2 Department of Orthopaedics, The Fifth Affiliated Hospital of Sun Yat-Sen University, Zhuhai, Guangdong, China; Faculty of Medicine and Biomedical Sciences, the University of Yaoundé I, Yaoundé, Cameroon, CAMEROON

## Abstract

**Background:**

Coffee is the most commonly consumed beverage among children and adolescences. Caffeine was demonstrated to be associated with bone metabolism. However, the relationship between caffeine intake and BMD in children and adolescents remains unclear. This study aimed to identified relationship between caffeine consumption and bone mineral density (BMD) in children and adolescents.

**Methods:**

Based on National Health and Nutrition Examination Survey (NHANES), we conducted an epidemiological cross-section study to measure the relationship between caffeine consumption and BMD in children and adolescents by multivariate linear regression models. Then, five methods of Mendelian randomization (MR) analyses were performed to estimate their causal relationship between coffee and caffeine intake and BMD in children and adolescents. MR-Egger and inverse-variance weighted (IVW) were used to evaluate the heterogeneity effect of instrumental variables (IVs).

**Results:**

In epidemiological studies, individuals with the highest quartile of caffeine intake do not have a significant change in femur neck BMD (β = 0.0016, 95% CI: -0.0096, 0.0129, *P* = 0.7747), total femur BMD (β = 0.0019, *P* = 0.7552), and total spine BMD (β = 0.0081, *P* = 0.1945) compared with the lowest quartile. In MR analysis, the IVW-random effect indicates no causal relationship between coffee consumption and TB- BMD (β = 0.0034, *P* = 0.0910). Other methods of MR analyses and sensitivity analysis reveals consistent findings. Similarly, the fixed-effects IVW method shows no causal association between caffeine intake and TB-BMD in children and adolescents (β = 0.0202, *P* = 0.7828).

**Conclusions:**

Our study does not support a causal relationship between caffeine consumption and BMD in children and adolescents. However, more studies are needed to verify our findings, such as its underlying molecular mechanisms and the long-term impact of early caffeine exposure at a younger age.

## Background

Osteoporosis (OP) is a bone disorder featured by decreased bone mass density (BMD) and impaired microarchitecture, causing bone pain and a higher risk of fragility fracture for the old [1]. The global prevalence of osteoporosis among adults aged 50–59, 60–69, and 70–79 was 11.4%, 24.8, and 37.6%, respectively [2]. In 2013, twenty-two million females and 5.5 million males in European Union (EU) countries suffered from osteoporosis, leading to 3.5 million fragility fractures annually [3]. The peak bone mass (PBM) in adolescence is a crucial factor in the process of OP and brittle fractures in the elderly [4]. A previous study found that a 10% PBM increase was linked to a 50% decrease in fragility fracture risk in older age [5]. Boreham et al. reported that a 6.4% reduction in PBM during childhood and adolescence could double the risk of fragility fractures in adulthood [6]. Studies have indicated that PBM formation could be influenced by genetics, diet and nutrition, physical activity, some diseases, and other factors [7–9].

Coffee is a trendy beverage worldwide, and approximately 73% of children drink caffeinated products daily [10]. Caffeinated soft drinks are a major source of caffeine for children and adolescents, containing between 50–500 mg of caffeine per bottle (equivalent to 5 cups of coffee) [11]. In recent decades, much speculation has been about the potential link between caffeine intake and osteoporosis. Many studies have been carried out to explore their relationship from the angle of clinical studies and molecular mechanism investigation [12–16]. Liu et al. [17] claimed in their study that caffeine could reduce BMD by enhancing osteoclastogenesis in rats. In addition, Rapuri et al. [18] proved that caffeine could impair osteoblast activation by reducing the vitamin D receptor expression on human osteoblasts and alkaline phosphatase activity. However, clinical research has always drawn inconsistent conclusions with different populations, study designs, or doses of caffeine intake [13, 19, 20]. A recent study of the National Health and Nutrition Examination Survey (NHANES) by Wang et al. [19] indicated caffeine could increase lumbar spine BMD in women aged 30–39 but could have a negative effect on men aged 40–49. In another study of young adult women aged 19–26, caffeine consumption was proven not to be a predictor of lower BMD [13]. Nevertheless, no studies investigate the relationship between caffeine consumption and BMD in children and adolescents [21]. Therefore, it is essential to uncover the relationship between caffeine consumption and BMD in children and adolescents as they obtain most of the PBM at the end of adolescence.

In order to fully evaluate associations between caffeine consumption and bone mineral density in children and adolescents, we conducted a cross-sectional study based on NHANES to determine the relationship between caffeine consumption and BMD in children and adolescents. Then, we implemented a two-sample Mendelian randomization (MR) analysis to evaluate their causal relationship at the genetic level. Two-sample MR is a novel method that uses a genetic variation of) to assess the causal relationship of exposure with outcome [22]. MR analysis could avoid confounding factors and infer causality since the alleles of exposure genetic variants are randomly assigned [23].

## Methods

### Ethics approval and consent to participate

The studies involving human participants were reviewed and approved by the NHANES Institutional Review Board. The use of data was approved by the ethics review board of the National Center for Health Statistics. The study procedures were structured in line with the Declaration of Helsinki. Written consent was obtained from all subjects and their legal guardian(s).

### Observational epidemiological analysis

NHANES project is an American national nutrition survey using a stratified, multi-stage random sampling design. We chose two cycles of NHANES (2007–2008, 2009–2010) since the data on BMD in children and adolescence existed in the two cycles. In the beginning, 4,264 subjects aged 8–19 were found in NHANES 2007–2008 and 2009–2010. After excluding 949 subjects without caffeine intake and 321 subjects without BMD (femur and lumbar spine BMD), 2,994 eligible subjects were included in our analysis. The flow chart of the research was exhibited in S1 Appendix.

### Variables included in the observational cross-sectional study

Based on two 24-hour dietary recall interviews, the individual’s caffeine intake was calculated by counting all caffeinated beverages and foods, including coffee, tea, soda and chocolate. The analysis used the average caffeine intake from two 24-hour recalls. We selected femur BMD (femur neck and total femur BMD) and lumbar spine BMD as the outcome variable in our analysis. Professionals collected and standardized BMD measured by Dual-energy X-ray absorptiometry (DXA) examinations. Covariates in the multiple regression analysis were chosen based on previous studies [8, 24, 25]. All data collection and processing were detailed at https://www.cdc.gov/nchs/nhanes/.

### Sources of two-sample MR

**Fig 1:**
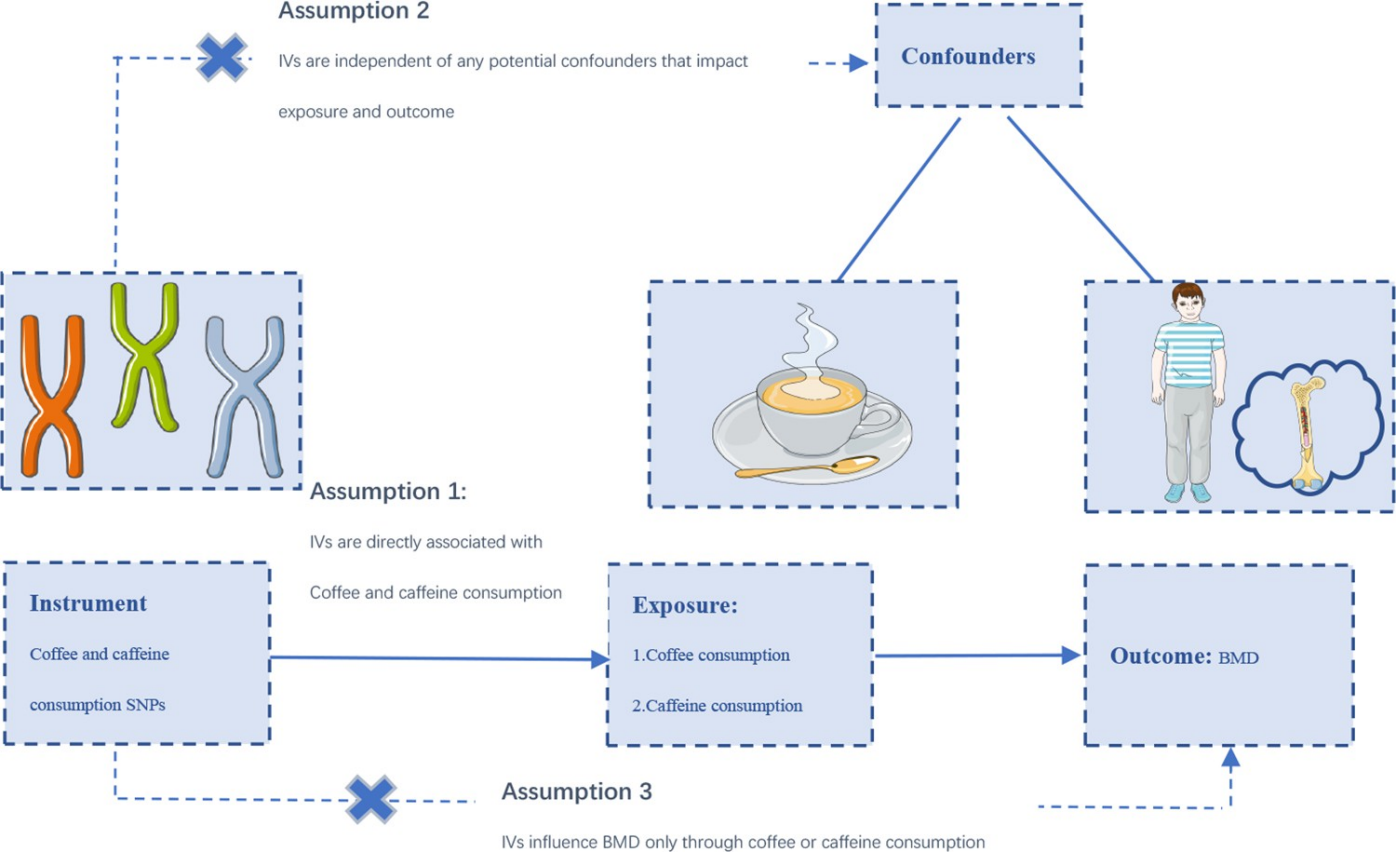
The study design of two‐sample MR analysis.

MR analysis was conducted based on three main assumptions (Fig 1) [26]. Fifteen SNPs strongly associated with coffee consumption were extracted from a meta-analysis of Genome-wide association studies (GWASs) on habitual coffee consumption [27], which adjusted for age, sex, BMI, total energy, and top 20 nutrients. In this genome-wide association study, over 370,000 adults of European ancestry were used to determine SNPs related to coffee consumption. Coffee consumption was available from assessment center visit and 24-h recalls. The study begins with a discovery analysis UK Biobank followed by replication in three independent US cohorts. Three SNPs (rs12699844, rs4719497, rs117692895) were excluded due to linkage disequilibrium (LD (r^2^> 0.01 and clumping distance <10,000kb). Finally, 12 independent SNPs were used as instrumental variables (IVs) for coffee consumption. Two SNPs closely related to caffeine consumption (*P*< 5E−8) were selected as IVs from a meta-analysis of GWAS (9,876 European descendants) [28]. Caffeine consumption was measured with caffeine-related metabolites in plasma and urine. The two SNPs explained about 1.31% of the variance in caffeine consumption.

Summary-level data for total body BMD (TB-BMD) of children and adolescents were obtained from a GWAS meta-analysis, which comprised 30 epidemiological studies of 11,807 individuals aged 0–15 years [29]. The GWAS meta-analysis was adjusted for age, weight, height, genomic principal components in a linear regression model.

### Statistical analysis

In observational epidemiological analysis, participants were grouped based on caffeine intake quartiles (quartile 1: 0–1.5 mg/d; quartile 2: 1.5–13.5 mg/d; quartile 3: 13.5–43.5 mg/d; quartile 4: >43.5 mg/d). We performed weighted chi-square tests for analyzing categorical variables and linear regression models for continuous variables. Multivariate linear regression models were performed to evaluate the relationship of caffeine intake (quartile) with femur and spine BMD. We built three models: model 1 (unadjusted model), model 2 (adjusting race/ethnicity, gender, age), and model 3 (adjusting race/ethnicity, gender, age, BMI, PIR, serum phosphorus, and serum calcium). A *P*-value less than 0.05 is considered statistical significance. MR is a method that uses genetic variation to estimate the causality between exposure and outcome. We employed five methods to examine the causal association between coffee consumption and TB-BMD in children and adolescents, including inverse-variance weighted (IVW), MR-Egger, weighted mode, weighted median, and simple model, among which IVW was the primary method [30]. Fixed-effects IVW (<3 SNPs) was performed to assess the genetic predictions between caffeine consumption and TB-BMD. The MR-Egger method and MR pleiotropy residual sum and outlier (MR-PRESSO) were used to test its horizontal pleiotropic. Outlier SNPs were detected by MR-PRESSO packages and then deleted. Cochran’s Q statistic was applied to examine the heterogeneity of individual SNPs in IVW and MR-Egger tests. Sensitivity analysis was performed by removing single SNP one by one. All data were analyzed by EmpowerStats software and R software.

## Results

### Epidemiological observation and analysis

Overall, 2,994 subjects were included in our analysis, with a mean age of 13.48 ± 3.32. Of these subjects, 6.27% are other Hispanic, 13.33% are Mexican American,13.46% are non-Hispanic black, 59.81% are non-Hispanic white, and 7.12% are other races (including multiracial population). Weighted baseline characteristics of all subjects were as described by quartiles of caffeine intake (Table 1). Then, we describe the baseline characteristics study population with caffeine consumption and no caffeine consumption. (S2 Appendix). Table 2 displays the results of the weighted multivariate regression analysis. In the unadjusted model, caffeine intake was positively correlated with total spine BMD, total femur BMD and Femur neck BMD (*P* for trend< 0.001). Nevertheless, no correlations were in the adjusted models (models 2 and 3). Individuals with the highest quartile of caffeine intake do not have a significant change in femur neck BMD (β = 0.0016, 95% CI: -0.0096, 0.0129, *P* = 0.77), total femur BMD (β = -0.0019, 95% CI: -0.0141, 0.0102, *P* = 0.7552), and total spine BMD (β = 0.0065, 95% CI: -0.0046, 0.0176, *P* = 0.2536) compared with the lowest quartile in the fully adjusted model (Table 2).

**Table 1:** Characteristics of the study population based on caffeine intake quartiles.

		Caffeine intake quartiles(mg/d)				
	Total	Q1 (0–1.5)	Q2 (1.5–13.5)	Q3 (13.5–43.5)	Q4 (>43.5)	*P* value
Number of subjects (n)	2994	742	747	756	749	
Age (years)	13.48 ± 3.32	12.91 ± 3.26	12.19 ± 3.11	13.12 ± 3.18	15.10 ± 2.96	<0.001
Gender (%)						0.176
Men	52.39	52.49	53.33	48.95	54.25	
Women	47.61	47.51	46.67	51.05	45.75	
Race/ethnicity (%)						<0.001
Mexican American	13.33	15.2	15.05	16.05	8.69	
Other Hispanic	6.27	6.19	6.44	8.08	4.83	
Non-Hispanic White	59.81	48.53	55.33	56.52	73.47	
Non-Hispanic Black	13.46	22.19	13.81	13.71	6.95	
Other Race (Including Multi-Racial)	7.12	7.89	9.37	5.65	6.06	
BMI	22.12 ± 5.47	21.93 ± 5.58	20.76 ± 5.01	22.12 ± 5.51	23.28 ± 5.45	<0.001
PIR	2.63 ± 1.63	2.65 ± 1.66	2.67 ± 1.64	2.55 ± 1.61	2.65 ± 1.61	0.51
Serum total calcium (mmol/L)	2.40 ± 0.06	2.40 ± 0.06	2.41 ± 0.05	2.40 ± 0.06	2.40 ± 0.07	0.013
Serum phosphorus (mmol/L)	1.41 ± 0.17	1.41 ± 0.16	1.43 ± 0.16	1.41 ± 0.17	1.39 ± 0.19	<0.001
Lumbar spine BMD (g/cm2)	0.85 ± 0.20	0.83 ± 0.20	0.79 ± 0.20	0.83 ± 0.20	0.93 ± 0.18	<0.001
Total femur BMD (g/cm2)	0.90 ± 0.19	0.89 ± 0.19	0.85 ± 0.18	0.89 ± 0.18	0.96 ± 0.18	<0.001
Femur neck BMD (g/cm2)	0.84 ± 0.17	0.82 ± 0.17	0.79 ± 0.16	0.83 ± 0.17	0.89 ± 0.16	<0.001

Mean ± SD for continuous variables: the P value was calculated by the weighted linear regression model. (%) for categorical variables

The *P* value was calculated by the weighted chi-square test. Abbreviation: BMD, bone mineral density. BMI, Body mass index. PIR, poverty income ratio

**Table 2:** The association between caffeine intake and BMD in children and adolescence.

		Total spine BMD (g/cm^2^)			Total femur BMD (g/cm^2^)			Femur neck BMD (g/cm^2^)	
	Model 1 β (95% CI) *P* value	Model 2 β (95% CI) *P* value	Model 3 β (95% CI) *P* value	Model 1 β (95% CI) *P* value	Model 2 β (95% CI) *P* value	Model 3 β (95% CI) *P* value	Model 1 β (95% CI) *P* value	Model 2 β (95% CI) *P* value	Model 3 β (95% CI) *P* value
Caffeine intake categories (mg/d)									
Q1 (0–1.5)	Reference	Reference	Reference	Reference	Reference	Reference	Reference	Reference	Reference
Q2 (1.5–13.5)	-0.0440 (-0.0646, -0.0234) <0.001	-0.0041 (-0.0166, 0.0084) 0.5212	0.0015 (-0.0098, 0.0129) 0.7890	0.0405 (-0.0600,	-0.0058 (-0.0195, 0.0078) 0.4027	0.0003 (-0.0121, 0.0127) 0.9622	-0.0343 (-0.0520, -0.0166) 0.0001	-0.0039 (-0.0170, 0.0092) 0.5572	0.0028 (-0.0086, 0.0143) 0.6289
				-0.0210) <0.0001					
Q3 (13.5–43.5)	0.0054 (-0.0151, 0.0258) 0.6058	-0.0017 (-0.0141, 0.0106) 0.7829	-0.0025 (-0.0137, 0.0088) 0.6657	0.0010 (-0.0183, 0.0204) 0.9165	0.0000 (-0.0136, 0.0136) 0.9989	-0.0009 (-0.0132, 0.0114) 0.8879	0.0048 (-0.0128, 0.0223) 0.5938	0.0045 (-0.0084, 0.0175) 0.4926	0.0035 (-0.0078, 0.0149) 0.5430
Q4 (>43.5)	0.1029 (0.0836, 0.1222) <0.0001	0.0081 (-0.0041, 0.0203) 0.1945	0.0065 (-0.0046, 0.0176) 0.2536	0.0760 (0.0578, 0.0943) <0.0001	-0.0000 (-0.0135, 0.0134) 0.9942	-0.0019 (-0.0141, 0.0102) 0.7552	0.0665 (0.0500, 0.0830) <0.0001	0.0038 (-0.0090, 0.0166) 0.5616	0.0016 (-0.0096, 0.0129) 0.7747
*P* for trend	<0.001	0.158	0.72	<0.001	0.786	0.359	<0.001	0.344	0.777

Model 1 unadjusted. Model 2 adjusted for age, gender, and race/ethnicity. Model3 adjusted for race/ethnicity, gender, age, BMI, PIR, serum phosphorus, and serum calcium. Abbreviation: BMD, bone mineral density. BMI, body mass index. PIR, poverty income ratio.

### Coffee consumption and TB-BMD

**Fig 2:**
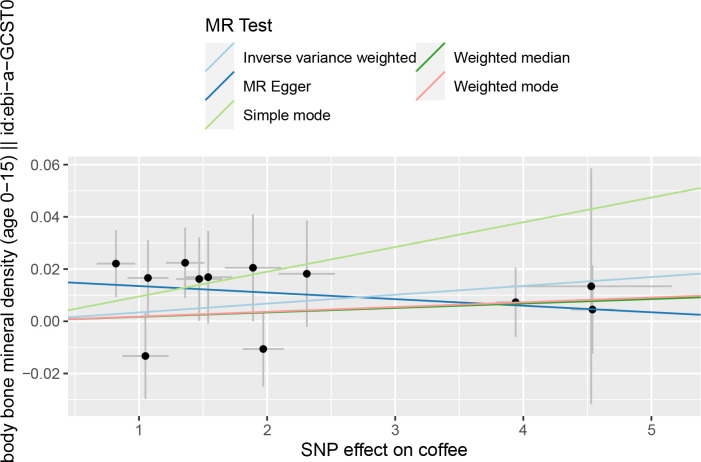
The scatter plot for MR analyses of causal associations between coffee consumption SNP and TB-BMD. Abbreviations: TB-BMD: total body bone mineral density. IVW: inverse-variance weighted.

**Fig 3:**
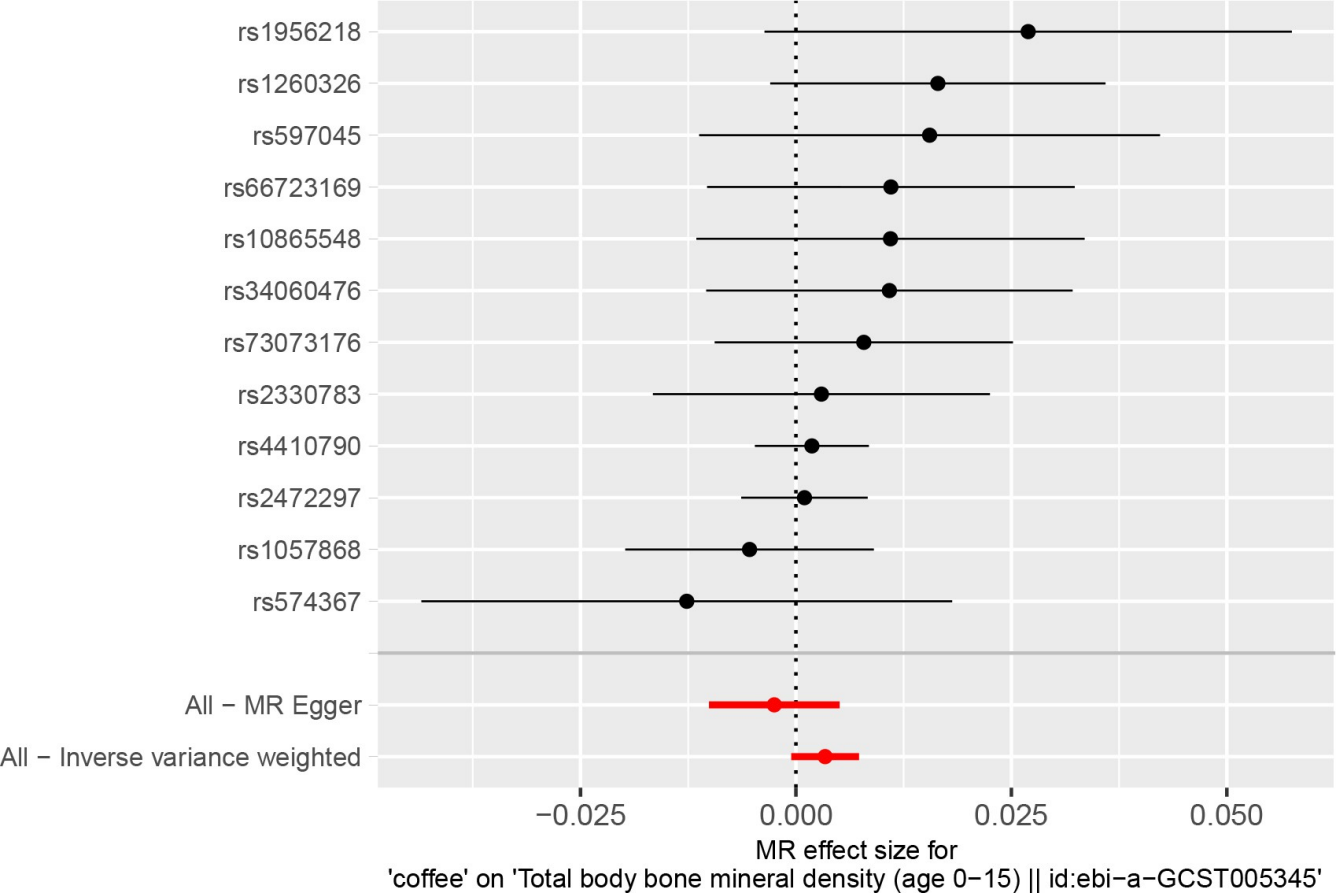
Forest plot to visualize causal effect of each single SNP of coffee consumption and TB-BMD.

Details on SNPs for caffeine and coffee consumption were summarized in Table 3. The result of the MR analysis was shown in Fig 2 and Table 4. The IVW results found no causal relationship between coffee consumption and the TB-BMD of children and adolescents (β = 0.0034, 95% CI: -0.0005, 0.0073, *P* = 0.0910). Similarly, no causal association was observed in the MR-Egger test, weighted mode, weighted median, and simple mode, either. The Cochran’s Q statistic of MR-Egger (Cochran’s Q = 6.362, *P* = 0.784) and IVW methods (Cochran’s Q = 9.546, *P* = 0.572) indicated no significant heterogeneity between IVs. There were no horizontal pleiotropic by MR-Egger regression (*P* = 0.105) and MR-PRESSO global test (*P* = 0.621). The forest plots of coffee consumption and TB-BMD were shown in Fig 3. The results were also consistent after the sensitivity analysis (S3 Appendix).

**Table 3:** Characteristics of SNPs associated with coffee and caffeine consumption.

						SNP-exposure (coffee and caffeine consumption)			SNP-outcome (TB-BMD)		
SNP	Nearby Gene	Chr	EA	OA	EAF	Beta	SE	*P*	Beta	SE	*P*
Coffee consumption											
rs1057868	POR	7	T	C	0.29	1.97	0.16	5.26E-33	-0.0106	0.0145	0.4669
rs10865548	TMEM18	2	G	A	0.83	1.54	0.19	4.46E-15	0.0169	0.0177	0.3401
rs1260326	GCKR	2	C	T	0.61	1.36	0.15	2.62E-19	0.0224	0.0135	0.0982
rs1956218	AKAP6	14	G	A	0.56	0.82	0.15	3.62E-08	0.0221	0.0128	0.0843
rs2330783	SPECC1L-	22	G	T	0.99	4.53	0.63	1.57E-12	0.0134	0.0452	0.7669
	ADORA2A										
rs2472297	CYP1A1/2	15	T	C	0.27	4.54	0.17	5.19E-155	0.0045	0.017	0.79
rs34060476	LOC101927630	7	G	A	0.13	1.89	0.22	5.06E-18	0.0205	0.0205	0.3157
rs4410790	AHR	7	C	T	0.63	3.94	0.15	5.59E-141	0.0073	0.0133	0.5828
rs574367	SEC16B	1	T	G	0.21	1.05	0.18	8.06E-09	-0.0133	0.0165	0.4215
rs597045	OR8U8	11	A	T	0.69	1.07	0.16	6.62E-11	0.0166	0.0146	0.256
rs66723169	MC4R	18	A	C	0.23	1.47	0.18	9.88E-17	0.0162	0.016	0.3108
rs73073176	MLXIPL	7	C	T	0.87	2.31	0.22	5.56E-25	0.0182	0.0204	0.3721
Caffeine consumption											
rs2470893	CYP1A1	15	T	C	0.31	0.12	0.016	5.15E-14	-0.005	0.0157	0.7485
rs4410790	AHR	7	C	T	0.62	0.15	0.017	2.36E-19	0.0073	0.0133	0.5828

*P* value < 5×10^−8^ for reporting genome-wide significance. Abbreviations: EA, effect allele. OA, other allele, EAF effect allele frequency. MR, Mendelian randomization. SE, standard error; SNP, single nucleotide polymorphism. TB-BMD, total body bone mineral density.

**Table 4:** Causal effect of coffee and caffeine consumption and TB-BMD.

Exposure	Outcome	Method	β	Lo CI	Up CI	*P*
Coffee consumption	TB-BMD of Children and adolescence	IVW	0.0034	-0.0005	0.0073	0.0910
		MR Egger	-0.0025	-0.0101	0.0051	0.5310
		Weighted median	0.0017	-0.0032	0.0066	0.4956
		Simple mode	0.0095	0.0009	0.0180	0.0523
		Weighted mode	0.0018	-0.0030	0.0068	0.4921
MR Egger: Cochran’s Q = 6.362, *P* = 0.784						
IVW: Cochran’s Q = 9.546, *P* = 0.572						
MR-Egger intercept = 0.016, *P* = 0.105						
MR-PRESSO global test = 0.621	TB-BMD of Children and adolescence	IVW	0.0202	-0.1236	0.1641	0.7828
Caffeine consumption						
IVW: Cochran’s Q = 6.362, *P* = 0.568						

No outlier was observed in the MR-PRESSO analysis in MR analysis in coffee consumption and TH-BMD. Abbreviations: CI, confidence interval; MR, Mendelian randomization

IVW, inverse-variance weighted; TB-BMD, total body bone mineral density.

### Caffeine consumption and TB-BMD

Similarly, the fixed-effects IVW analysis suggested that there was no causal effect of caffeine intake on TB-BMD of children and adolescents (β = 0.0202, 95% CI: -0.1236, 0.1641, *P* = 0.7828) (Table 4). IVW methods showed no significant heterogeneity existing (Cochran’s Q = 6.362, *P* = 0.568). MR-PRESSO and MR-Egger intercept tests could not be performed because of the absence of sufficient IVs.

## Discussion

This work employed a large observational epidemiological analysis and a two-sample MR analysis to explore the association between caffeine intake and BMD in children and adolescents. Our study found no causal association between caffeine intake and BMD, providing novel evidence for caffeine’s potential roles in the bone health of children and adolescents.

Caffeine is very common in the diet of people worldwide. Soda, tea, specialty coffee drinks, and food products like candy bars, potato chips, and gum [31] contain caffeine. Over the past few decades, there has been a keen interest in exploring the impact of these popular foods on human health [32–34]. Extensive studies demonstrated that caffeine was related to a decreased risk of all-cause mortality, Parkinson’s disease, liver diseases, cardiovascular mortality, type 2 diabetes, and so on [35, 36]. However, the association between caffeine intake and BMD or osteoporosis remains controversial from molecular studies and epidemiological studies of adult or elderly populations only [37–39]. In a prospective study from Taiwan between 2006 and 2014, 2,682 Taiwanese aged 30 years and older were included in the analysis [37]. Each completed questionnaire included weekly coffee consumption frequency, which was identified by asking subjects about the frequency of coffee drinking per week. BMD was evaluated by quantitative ultrasound (QUS) at the calcaneus [37]. As a result, caffeine consumption was positively linked with T-scores for both sexes. In another cross-sectional study of postmenopausal women in Korea, individuals with the highest caffeine consumption quartile were at a lower risk of osteoporosis than individuals in the lowest quartile (OR = 0.64; 95%CI, 0.43–0.95). This finding was consistent in osteoporosis of the femoral neck and lumbar spine (OR = 0.55 and 0.65, respectively). In addition, coffee intake was positively related to the lumbar spine and femoral neck BMD. By contrast, a Swedish longitudinal study of 61,433 females showed that high coffee consumption (≥4 cups per day) was linked to a 4% reduction in BMD compared with low consumption (<1 cup daily). However, it did not increase the fracture risk (OR = 0.99; 95%CI, 0.98–1.00) [38]. In an early study, Barrett-Connor et al. [40] found that a lifetime intake of two cups of caffeinated coffee per day is associated with decreased BMD in older women. However, for daily milk drinkers, coffee-related osteoporosis was offset. In another study of young adult women aged 19–26, caffeine consumption was not related to BMD after adjusting age, height, BMI, calcium and protein intake, and alcohol use in linear regression models. In conclusion, the evidence of caffeine consumption and bone health was always inconsistent, However, clinical studies evaluating the effects of caffeine intake on BMD in children and adolescents were few. Our study using MR analysis which could avoid confounding factors and infer causality, indicates no causal association between caffeine intake and BMD, providing novel evidence for caffeine’s possible roles in the bone health of children and adolescents.

In vitro and animal studies, caffeine has been proven to exert biological effects that may affect bone metabolism and BMD. Evidence has shown that a major mechanism of caffeine in organs is its direct and indirect effects on bone metabolism through competitive inhibition of four adenosine receptors, including A1, A2A, A2B and A3 adenosine, which are expressed in undifferentiated osteoblast precursors and differentiated osteoblasts [41, 42]. Bone metabolism appears to be significantly regulated by A2A and A2B receptors. Mediero et al. [43] found that reduced extracellular adenosine levels can lead to decreased bone mass in ovariectomized mice, and A2B and A2A agonists can reverse this effect. In vitro and animal models, antagonism of A2A receptors influenced by caffeine promotes osteoclast formation and function, while antagonism of A2B receptors inhibits osteoblast formation [41]. In addition, caffeine blockade of A1 receptors can lead to increased osteoblast activity, in contrast to the pro-osteoporotic effect of caffeine on A2 receptors [44]. Thus, the effect of caffeine on bone metabolism depends on its ability to block A1 and A2 receptors and the importance of the affected signaling pathways. Furthermore, caffeine could disrupt calcium metabolism and alter the vitamin D receptor (VDR) to regulate bone metabolism, reducing bone mass in mice [18, 39]. In conclusion, at the molecular level, the conflicting effects of caffeine on A1 versus A2a and A2b receptors and which one predominates remain to be elucidated. Therefore, the multiple effects of caffeine on bone metabolism remain to be investigated by further molecular mechanisms and clinical research.

Our study has some advantages. First, the caffeine consumption in the cross-sectional study was based on average caffeine intake from two 24-hour recalls, which makes our results more reliable. Second, in addition to epidemiological observation, MR analysis was conducted to examine their causal association. The results of the five methods of MR analysis were consistent, which added to the evidence of our research. Third, the MR analysis’s overall heterogeneity was low, verified by MR-Egger and IVW methods. No horizontal pleiotropic was found by MR-Egger regression and MR-PRESSO global test. In addition, the results were also consistent after the sensitivity analysis. However, some potential limitations could not be avoided. First, there may be personal questionnaire survey bias when recalling caffeine consumption because of the nature of questionnaire survey. In addition, other confounding factors such as hormonal status, medication use, and genetic factors were not included in linear regression models because of the data limitation. Second, we only took children older than eight into account since the limitation of the NHANES database. More research should be carried out to investigate the relationship between caffeine intake and BMD in children under 8 years old. Third, we found only one GWAS of BMD in children and children and adolescents. Furthermore, some SNPs related to coffee consumption were excluded due to linkage disequilibrium. However, the exclusion of certain SNPs due to linkage disequilibrium may have affected the power of the MR analysis. Further MR analysis based on updated GWASs is required to verify the results of our study. Fourth, the current MR study used data from GWAS meta-analysis with a predominantly European population. Whether the findings of this study can be generalized to other populations remains to be looked at, and more studies involving other races and populations are needed in the future. Fifth, due to the nature of epidemiology, the study may contain residual errors, such as measurement error. So, the result of this study should be interpreted with caution.

## Conclusions

Based on the cross-sectional study and Mendelian randomization study, our study does not support a causal association between caffeine consumption and BMD in children and adolescents. However, more studies with more robust evidence are needed to verify our findings, such as its underlying molecular mechanisms and the long-term impact of early caffeine exposure at a younger age.

## Supporting information

S1 Appendix Flowchart identifying process of the NHANES participants inclusion and exclusion. (PDF)

S2 Appendix Characteristics of study population with no caffeine consumption and caffeine consumption. (DOCX)

S3 Appendix Leave-one-out sensitivity analysis of MR analysis for coffee consumption and TB-BMD. (PDF)

## List of abbreviations

BMD: Bone mineral density
OP: Osteoporosis
NHANES: National Health and Nutrition Examination Survey
MR: Mendelian randomization
TB-BMD: Total body BMD
IVs: Instrumental variables
SNPs: Heterogeneous single-nucleotide polymorphisms
MR-PRESSO: MR pleiotropy residual sum and outlier
IVW: Inverse-variance weighted
EU: European Union
PBM: Peak bone mass
GWASs: Genome-wide association studies
QUS: Quantitative ultrasound
VDR: Vitamin D receptor
