# Supplementary figures and images for: Association between caffeine consumption and bone mineral density in children and adolescent: Observational and Mendelian randomization study
Source: PLoS One. 2023 Jun 29;18(6):e0287756. doi: 10.1371/journal.pone.0287756 (PMC10309635; doi:10.1371/journal.pone.0287756)

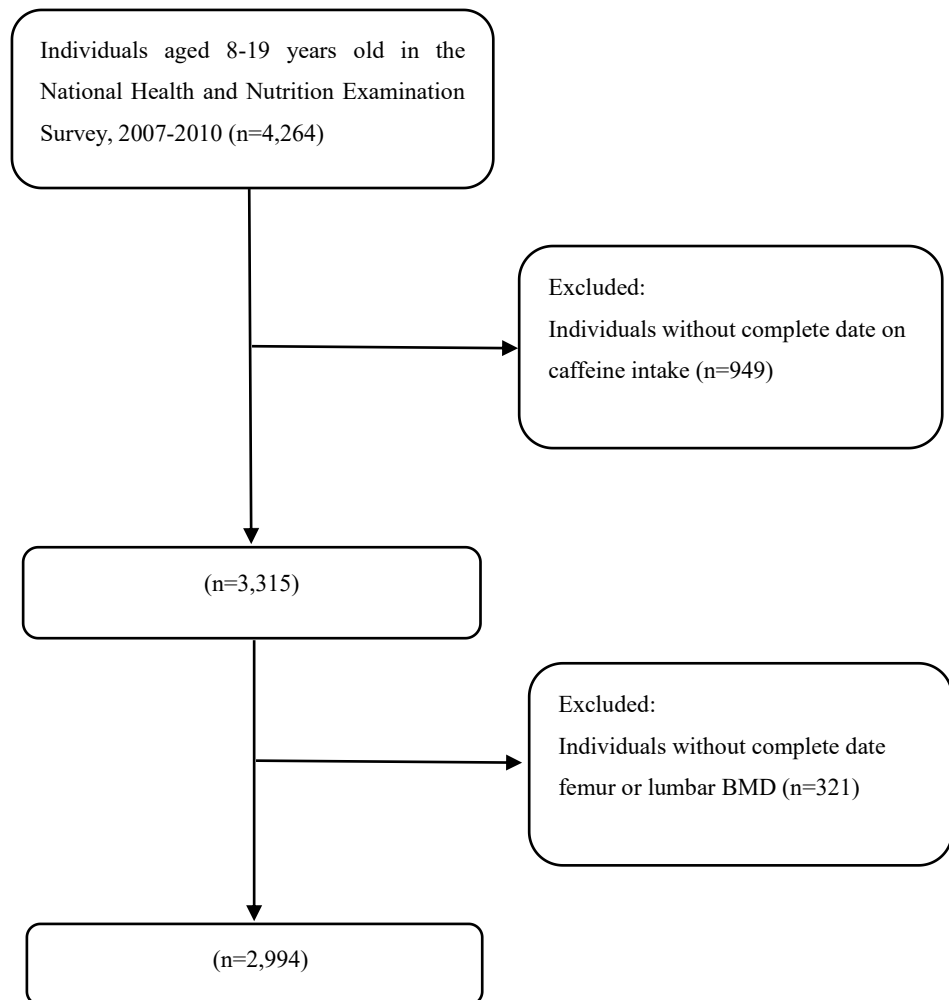

Supplement: S1 Appendix — (PDF) [file pone.0287756.s001.pdf]
