# Supplementary material for: Association between caffeine consumption and bone mineral density in children and adolescent: Observational and Mendelian randomization study
Source: PLoS One. 2023 Jun 29;18(6):e0287756. doi: 10.1371/journal.pone.0287756 (PMC10309635; doi:10.1371/journal.pone.0287756)
Supplement: S2 Appendix — (DOCX) [file pone.0287756.s002.docx]

**Appendix 2**: Characteristics of study population with no caffeine consumption and caffeine consumption.

|  | No caffeine consumption group | Caffeine consumption group | *P* value |
| --- | --- | --- | --- |
| Number of subjects (n) | 449 | 2545 |  |
| Age (years) | 13.05 ± 3.32 | 13.13 ± 3.42 | 0.643 |
| Gender (%) |  |  | 0.834 |
| Men | 234 (52.12%) | 1340 (52.65%) |  |
| Women | 215 (47.88%) | 1205 (47.35%) |  |
| Race/ethnicity (%) |  |  | <0.001 |
| Mexican American | 120 (26.73%) | 682 (26.80%) |  |
| Other Hispanic | 50 (11.14%) | 326 (12.81%) |  |
| Non-Hispanic White | 85 (18.93%) | 883 (34.70%) |  |
| Non-Hispanic Black | 164 (36.53%) | 510 (20.04%) |  |
| Other Race (Including Multi-Racial) | 30 (6.68% | 144 (5.66%) |  |
| BMI | 22.79 ± 6.10 | 22.20 ± 5.60 | 0.043 |
| PIR | 2.11 ± 1.51 | 2.10 ± 1.51 | 0.884 |
| Serum total calcium (mmol/L) | 2.40 ± 0.06 | 2.40 ± 0.06 | 0.375 |
| Serum phosphorus (mmol/L) | 1.41 ± 0.16 | 1.41 ± 0.16 | 0.625 |
| Lumbar spine BMD (g/cm2) | 0.84 ± 0.21 | 0.84 ± 0.20 | 0.572 |
| Total femur BMD (g/cm2) | 0.91 ± 0.19 | 0.89 ± 0.19 | 0.196 |
| Femur neck BMD (g/cm2) | 0.84 ± 0.17 | 0.83 ± 0.17 | 0.251 |

**Mean ± SD for continuous variables: the P value was calculated by the weighted linear regression model. (%) for categorical variables. The *P* value was calculated by the weighted chi-square test.** **Abbreviation: BMD, bone mineral density. BMI, Body mass index. PIR, poverty income ratio**
