# Supplementary material for: Association between caffeine consumption and bone mineral density in children and adolescent: Observational and Mendelian randomization study
Source: PLoS One. 2023 Jun 29;18(6):e0287756. doi: 10.1371/journal.pone.0287756 (PMC10309635; doi:10.1371/journal.pone.0287756)
Supplement: S3 Appendix — (PDF) [file pone.0287756.s003.pdf]

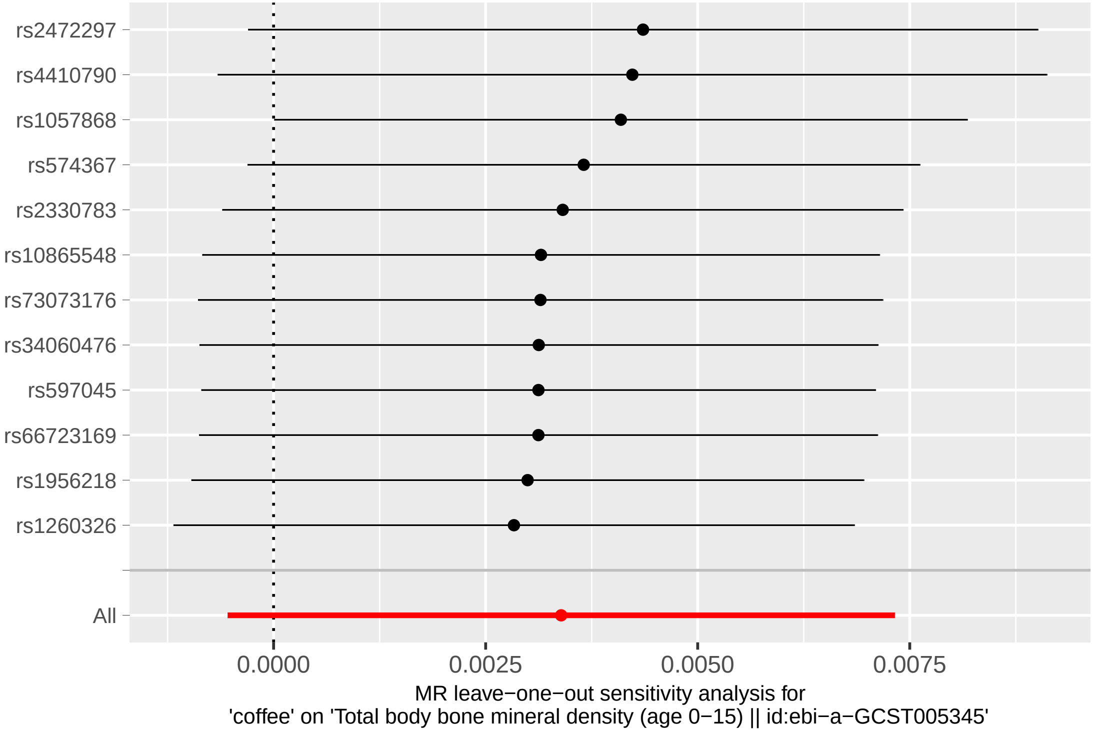
rs2472297
rs4410790
rs1057868
rs574367
rs2330783
rs10865548
rs73073176
rs34060476
rs597045
rs66723169
rs1956218
rs1260326
All
0.0000
0.0025
0.0050
0.0075
MR leave−one−out sensitivity analysis for
'coffee' on 'Total body bone mineral density (age 0−15) || id:ebi−a−GCST005345'
